# Prevalence and Severity of Burn Scars in Rural Mozambique

**DOI:** 10.1007/s00268-022-06682-y

**Published:** 2022-08-10

**Authors:** Patrick Barba, Daniel C. Neubauer, Matchecane Cossa, Jeremy Sieker, Michael W. Hornacek, Samuel H. Lance, Emily Ewing, Catherine Tsai, Carlos Funzamo, Vanda Amado, Fatima Adamo, John Rose, Peter Bendix, Fernando Vaz, Emilia Noormahomed, Stephen W. Bickler, Amanda Gosman

**Affiliations:** 1 School of Medicine, University of California San Diego, 9500 Gilman Dr, La Jolla, CA USA; 2 Department of Surgery, Division of Plastic Surgery, University of California San Diego, San Diego, CA USA; 3 Department of Surgery, Eduardo Mondlane University, Maputo, Mozambique; 4 Rady Children’s Hospital, University of California, San Diego, CA USA; 5 Department of Surgery, University of California San Diego, San Diego, CA USA; 6 Department of Surgery, Division of Plastic and Reconstructive Surgery, University of California San Francisco, San Francisco, CA USA; 7 Department of Surgery, University of Chicago, Chicago, IL USA; 8 Department of Parasitology, Eduardo Mondlane University, Maputo, Mozambique; 9 Division of Pediatric Surgery, Rady Children’s Hospital, University of California, San Diego, CA USA

## Abstract

**Background:**

Burn injuries are common in low- and middle-income countries (LMICs) and their associated disability is tragic. This study is the first to explore burn scars in rural communities in Mozambique. This work also validated an innovate burn assessment tool, the Morphological African Scar Contractures Classification (MASCC), used to determine surgical need.

**Methods:**

Using a stratified, population-weighted survey, the team interviewed randomly selected households from September 2012 to June 2013. Three rural districts (Chókwè, Nhamatanda, and Ribáuè) were selected to represent the southern, central and northern regions of the country. Injuries were recorded, documented with photographs, and approach to care was gathered. A panel of residents and surgeons reviewed the burn scar images using both the Vancouver Scar Scale and the MASCC, a validated visual scale that categorizes patients into four categories corresponding to levels of surgical intervention.

**Results:**

Of the 6104 survey participants, 6% (*n* = 370) reported one or more burn injuries. Burn injuries were more common in females (57%) and most often occurred on the extremities. Individuals less than 25 years old had a significantly higher odds of reporting a burn scar compared to people older than 45 years. Based on the MASCC, 12% (*n* = 42) would benefit from surgery to treat contractures.

**Conclusion:**

Untreated burn injuries are prevalent in rural Mozambique. Our study reveals a lack of access to surgical care in rural communities and demonstrates how the MASCC scale can be used to extend the reach of surgical assessment beyond the hospital through community health workers.

**Supplementary Information:**

The online version contains supplementary material available at 10.1007/s00268-022-06682-y.

## Introduction

Burns are a significant cause of preventable morbidity and mortality in sub-Saharan Africa [1]. Low-income countries, like Mozambique, disproportionately bear the burden of burns and may have inadequate resources to treat them [2]. Burn injuries are most common in children under 12 years old and limited access to surgical care negatively impact the outcomes of these injuries [3, 4] While incidence and severity of burns has been explored in emergency room admissions to large hospitals in different sub-Saharan African locations, the surgical burden of burn injuries outside these large centers is not well characterized [5, 6]. Community-level research is necessary to capture this information and inform the medical community of the true extent of burn injuries [7].

The World Health Organization (WHO) notes that burns are among the leading cause of daily-adjusted life years (DALYs) lost in low- and middle-income countries [8]. Roughly 180,000 deaths per year are attributed to burns with the majority occurring in sub-Saharan Africa. The Institute for Health Metrics and Evaluation estimates 172.07 per 100,000 DALYs lost because of fire, heat, and hot substances in Mozambique in 2019 [9]. Beyond mortality, burn injuries often require long hospital stay and can lead to debilitating long-term effects [10]. In areas where access to medical care is limited, burn survivors are forced to carry the significant physical and psychological burden these associated with these injuries [11]. Burn location is an important influence on management and long-term outcomes [12]. Burn injuries and the resulting scars, especially near joints or in the distal extremities, can lead to significant physical limitations and occasionally cause a permanent disability [13].

Scoring scars and qualifying the need for surgical intervention is vital to accurately assess need. Though multiple rating scales to assess burn scars exist in the literature, most require a comprehensive physical exam and standardized photographs to be effective [14, 15]. These data and the corresponding photographs gathered by community health workers in a large-scale survey require a simpler rating scale that can accurately reflect surgical need without necessitating complex techniques and expensive equipment.

This study sought to quantify the prevalence of burn scars in three rural communities in Mozambique, qualify these injuries with a novel scar scale, and determine the proportion amenable to surgical intervention.

## Methods

### Data collection

**Fig. 1:**
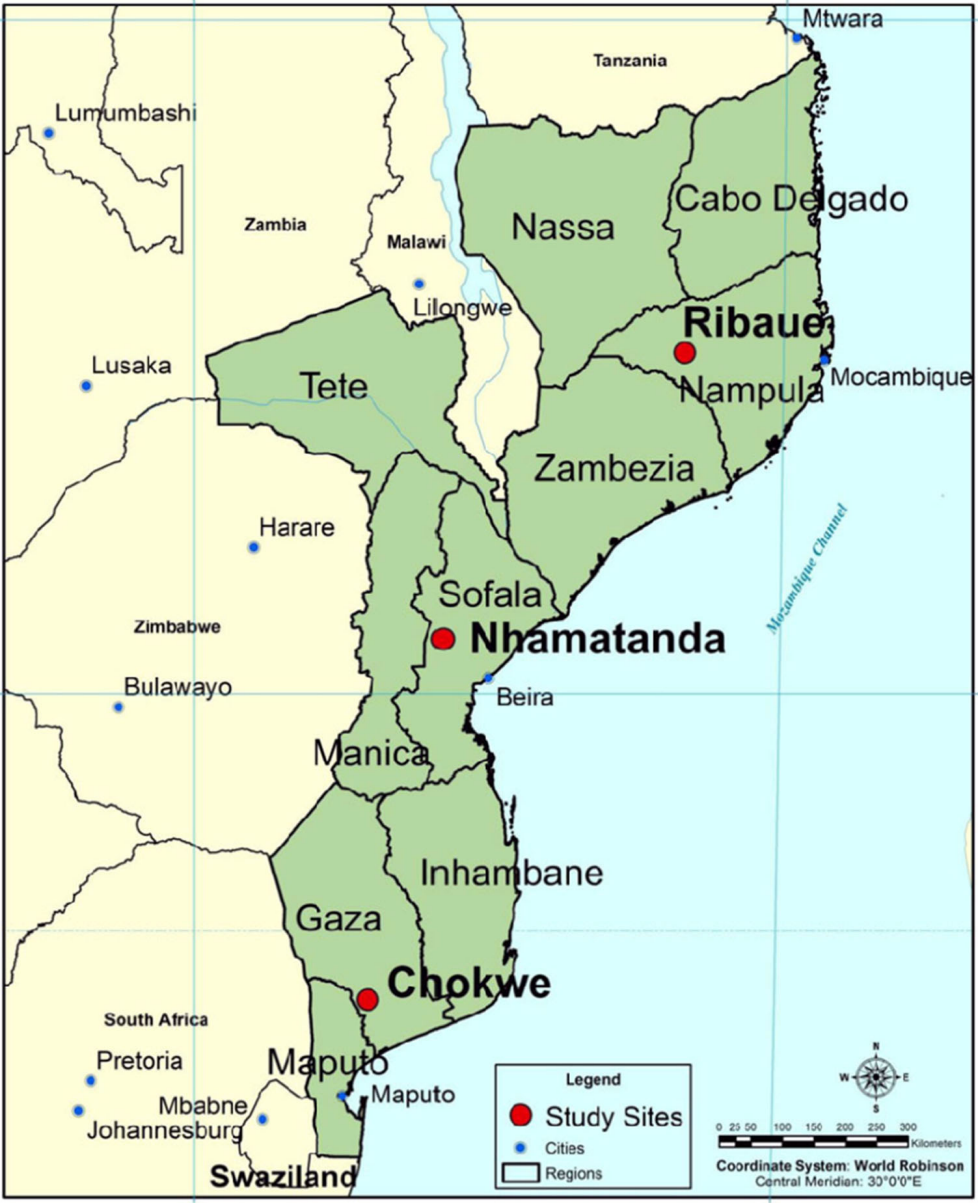
Map of three rural study sites in Mozambique adapted from Anderson et al. [21]

Approval was obtained from the UCSD Human Research Protection Program and the National Bioethics Committee in Mozambique. The research team queried a database outlined by Rose et al., who gathered a stratified, population-weighted household survey in three rural districts (Chókwè, Nhamatanda, and Ribáuè) of Mozambique in 2012 and 2013 (Fig. 1) [16]. They were chosen to represent the southern, central and northern regions of the country [16]. Each member of the selected households was surveyed and injuries were coded into six distinct anatomical areas: face/head/neck, chest/breast, back, abdomen, groin/genitals/buttocks, and extremities. Approach to treatment were also gathered. Photographs of the relevant condition were taken with a smartphone, coded to protect patient identity and linked to the individual’s responses. These data were stored on a password-protected server in a cloud storage environment. (Dropbox Inc. San Francisco, CA, USA).

### Primary analysis

All coded burns injuries were factored into overall prevalence, but those that were not matched with a photograph were removed from secondary data analysis because their anatomical location could not be verified.

### Coding

Burn photographs were reclassified into the correct anatomical area when necessary. The Surgeons OverSeas Assessment of Surgical Need (SOSAS) guidelines were adapted for the reclassifications [17]. Burns that crossed joints were flagged for separate analysis.

### Secondary analysis

A panel of general surgery residents and plastic surgeons reviewed the images using the Morphological African Scar Contractures Classification (MASCC) [18]. This scale uses three metrics, length, width and height compared to the joint a burn scar occupies, to categorize burns by the complexity of the surgery needed for their correction (Supplemental Material). The same panel of residents and surgeons also scored the images using the Vancouver Scar Scale, an established burn scar rating metric, to validate this novel tool [19, 20].

### Statistical analysis

Descriptive statistics including age, gender, burn anatomical location and approach to care were calculated. Tables and plots were generated with divisions by age group and burn location. Statistical analysis was performed in R (Version 1.3.1093, RStudio Team (2020). RStudio: Integrated Development for R. RStudio, PBC, Boston, MA) using odds ratios to assess different sub-groups. Inter-rater reliability and inter-class correlation was calculated for the Vancouver Scar Scale, and Cohen’s Kappa was calculated for the categorical MASCC.

## Results

Of the 6104 total survey respondents, 2383 (39%) were from Chókwè in the south, 1657 (27%) were from Nhamatanda in the central region, and 2064 (34%) were from Ribáuè in the north. There were slightly more females (3300, 54%) than males, and 22% of the total respondents were under 15 years of age.

A total of 370 (6%) individuals reported one or more burn injuries. Of the 370 individuals who reported a burn injury, 209 (57%) were female and 161 (43%) were male, but this observed difference was not statistically significant. As shown in Table 1, the age groups reporting the highest proportion of burns were 15–24 years (107, 8.22%), followed by the 5–14 years (68, 7.61%). Compared to individuals over 45 years of age, individuals in the age ranges 0–4, 5–14, and 15–24 all had statistically higher odds of reporting a burn scar. Of the 370 individuals reporting burns, 19 (5%) reported burns in multiple anatomical areas. After review of the data gathered, the accompanying photographs were used to verify anatomical location and recoded when necessary. A total of 47 individuals were excluded due to missing or inadequate photographs of the burns.

**Table 1:** Demographics of patients with burn scars in three rural areas of Mozambique (*N* = 370)

	Total	Burns *n* (%)	Odds Ratio (95% CI)
Sex			
Male	2804	161 (5.74)	Reference
Female	3300	209 (6.33)	1.11 (0.90, 1.37)
Age			
0–4 years	434	34 (7.83)	2.05 (1.32, 3.19)*
5–14 years	893	68 (7.61)	1.99 (1.39, 2.86)*
15–24 years	1302	107 (8.22)	2.16 (1.56, 3.01)*
25–45 years	2040	104 (5.10)	1.30 (0.93, 1.81)
> 45 years	1435	57 (3.97)	Reference
Median age (range)	23 (1–89)		

Data are *n*/*N* (%) unless otherwise specified. *P* values < 0.05 noted with *

*OR* Odds ratio; *CI* Confidence interval

**Fig. 2:**
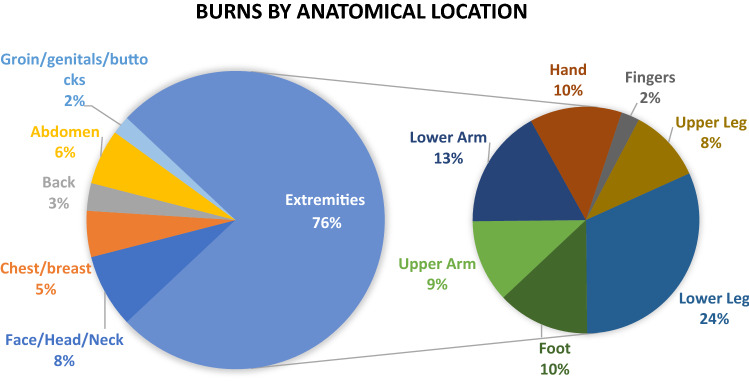
Burn Scars by Anatomical Location (*n* = 347)

**Fig. 3:**
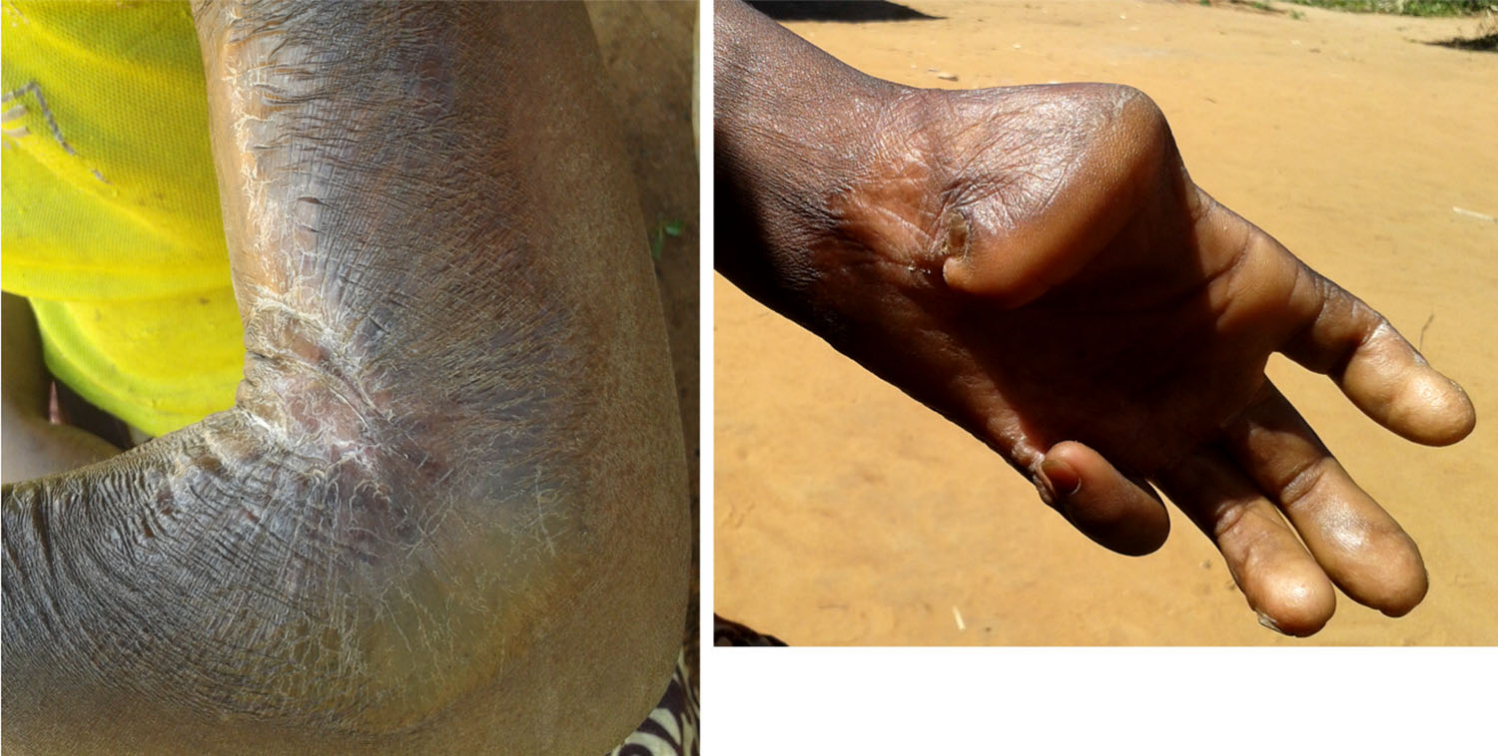
Disfiguring burns that could benefit from surgical correction

Accounting for this, a total of 322 individuals reported 347 unique burn injuries. As seen in Fig. 2, most of these injuries occurred in the extremities (76%) followed by the face/head/neck (8%) and the abdomen (6%). Burns in the extremity were most often reported on the lower leg. Thirty-five burns (10%) crossed a joint (Fig. 3). Regarding the timing of these injuries, 262 burns (76%) occurred more than 1 year prior, while the remaining 85 (24%) occurred within 1 year.

Table 2 shows the respondents’ approaches to seeking and receiving healthcare. Sixty-five percent of respondents (227) with burns sought healthcare at a facility. Most received dressings (203, 59%) and 16 (5%) received surgery. Of the minority that did not receive formal healthcare (105, 30%), “No need” was the most common response (42, 12%) followed by “No money for healthcare” (19, 5%). Fourteen respondents (4%) went to a traditional healer. Gender did not significantly affect the odds of seeking and receiving healthcare. Age and burn location significantly affected the odds of seeking healthcare, and burns crossing a joint were significantly more likely to receive surgery compared to burns that did not involve a joint (Table 2).

**Table 2:** Approach to burn care stratified by sex, crossing a joint, age and burn location (*n* = 347)

	Health care sought			Health care received		
	Yes	No	OR (95% CI)	Dressings	Surgery	OR (95% CI)
Sex						
Male	94	49	Ref	79	9	Ref
Female	132	72	0.96 (0.61, 1.50)	116	7	1.89 (0.68, 5.28)
Cross Joint						
Yes	32	3	Ref	23	5	Ref
No	194	118	0.15 (0.05, 0.51)*	172	11	3.40 (1.08, 10.66)*
Age						
0–4 years	23	8	2.78 (1.07, 7.23)*	23	0	N/A
5–14 years	48	21	2.21 (1.06, 4.58)*	45	2	2.70 (0.42, 17.25)
15–24 years	63	33	1.84 (0.94, 3.60)	55	5	1.32 (0.29, 5.96)
25–45 years	63	28	2.17 (1.10, 4.31)*	47	6	0.94 (0.22, 4.08)
> 45 years	29	28	Ref	25	3	Ref
Burn Location						
Face	34	6	Ref	27	6	Ref
Chest	32	20	0.28 (0.10, 0.79)*	27	1	6.00 (0.68, 53.25)
Back	30	6	0.88 (0.26, 3.03)	24	3	1.78 (0.40, 7.90)
Abdomen	31	11	0.50 (0.16, 1.51)	22	5	0.98 (0.26, 3.64)
Groin	7	1	1.24 (0.13, 11.93)	6	1	1.33 (0.13, 13.22)
nExtremities	182	101	0.32 (0.13, 0.78)*	156	12	2.89 (1.00, 8.35)

*OR* Odds ratio; *CI* Confidence Interval *P* values < 0.05 noted with *

Based on the scale adapted from the MASCC, 42 (12%) burns would benefit from surgical correction to treat contractures. These burns had an average score on the VSS of 6.45 (IQR = 5–8). Most burn scars that could benefit from surgical correction are in the extremities and only five individuals (11.9%) had prior surgery (Table 3). Type D burns, characterized by long scars that occupy a larger portion of the related joint, were the most common across all raters, accounting for 53% of the surgical burns on average (Table 4). Interclass correlation (ICC) across all raters for the VSS was 0.951 (95% CI 0.923 – 0.971, *p* =  < 0.001), and remained high when comparing within resident raters (ICC = 0.889, 95% CI 0.816 –0.937, *p* =  < 0.001) and attending raters (ICC = 0.991, 95% CI 0.986–0.995, *p* =  < 0.001) (Table 5). The MASCC showed moderate agreement between all raters (*k* = 0.421, 95% CI 0.365–0.478, *p* =  < 0.001) and strong agreement between resident raters (*k* = 0.754, 95% CI 0.633–0.875, *p* =  < 0.001). Across both scar scales, Type C and D burns had a higher average VSS score than their Type A and B counterparts (6.6 vs 5.6, *p* = 0.03). There was no significant difference in rating between the resident and attendings physician scorers.

**Table 3:** Demographics and approach to care of patients with burns scars that could benefit from surgical correction (*n* = 42)

Characteristic	Burns *n* (%)
Sex	
Male	19 (45.24)
Female	23 (54.76)
Location	
Upper extremity	22 (52.38)
Lower extremity	12 (28.57)
Face/head/neck	5 (11.90)
Chest	2 (4.76)
Buttocks	1 (2.38)
Care sought	
Dressings	23 (54.76)
Surgery	5 (11.90)
Median age (range)	28.5 (4–69)

Data are *n*/*N* (%) unless otherwise specified

**Table 4:** Breakdown of Morphological African Scar Contracture Classification and Algorithm (MASCC) burn scar types by rating group (*n* = 42)

Raters average by burn type	Type A average (%)	Type B average (%)	Type C average (%)	Type D average (%)
Residents	7.3 (17.46)	1.3 (3.17)	24.7 (58.73)	8.7 (20.63)
Attendings	5 (11.90)	0.3 (1.03)	19.7 (46.83)	17 (40.48)
Overall	6.2 (14.68)	0.8 (2.05)	22.2 (52.86)	12.8 (30.48)

Average number of burn types and the related percentage values are shown for each rater group for the total

**Table 5:** Comparison of the Vancouver scar scale and morphological African Scar Contracture Classification and algorithm (*n* = 42)

	All raters	Significance	Resident raters	Significance	Attending raters	Significance
Vancouver scar scale						
Interclass correlation	0.951	< 0.001	0.889	< 0.001	0.991	< 0.001
MASCC						
Cohen’s Kappa	0.421	< 0.001	0.754	< 0.001	0.473	< 0.001

## Discussion

This study is the first of its kind to focus on the prevalence of burn scars in rural communities in Mozambique. The prevalence of burn scars as characterized by previous rural household surveys is between 6% (607 per 10,000) in Ghanaian children, and 4% (398 per 10,000) in Sierra Leone, which is similar to the prevalence found in this study of 6% (607 per 10,000) [10, 22]. While past community-based studies have focused on broadly-defined surgically correctable conditions, this work gathers individuals’ approach to care, photographs of relevant injuries, and is one of the first to focus exclusively on burn scars [23–25]. Using World Bank rural population estimates, there were an estimated 1.03 million individuals with burn scars in rural Mozambique in 2013 [26]. Using our findings that more than 12% of burn scars could benefit from surgical correction, this estimates that more than 130,000 individuals across rural Mozambique could benefit from corrective burn scar surgery.

This study shows that burns scars are common in rural Mozambique. While it has been documented that women are at an increased risk of sequelae from burn injuries, our data show no significant difference between men and women in healthcare sought or received [27–29]. Conversely, age and burn location have a significant influence on healthcare seeking behavior. Individuals ages 0–4 years, 5–14 years, and 25–45 years sought care at a significantly higher rate than individuals older than 45 years of age. Similarly, individuals reporting a burn to the face, head or neck were more likely to seek treatment than those who reported a burn to the chest or extremities. While most burns occurred more than 1 year before the time of the survey, one quarter of the burn injuries occurred within the year preceding the study. In addition to surgical correction of chronic burn scars, this highlights the importance of burn prevention strategies, such as safe cooking practices, in global burn care. In line with past reports in the literature, this study found that traditional healers do not play a large role in burn care [30].

There is limited access to safe surgical care in many LMICs [2, 31, 32]. Past work has cited burn depth to be the most important indication for surgery, but this work suggests the additional importance of joint involvement to the treatment paradigm [31]. In this study, burns that did not cross a joint were significantly more likely to be treated with dressings instead of a surgical procedure. Burns involving joints and the resulting contractures can severely limit range of motion and negatively impact function [12]. In rural communities such as those surveyed in this study, burns that involve joints should be carefully assessed for the need for surgical care to avoid potential long-term sequelae.

The adapted MASCC used in this study is an effective scale to qualify and rate burn scars by the complexity of the surgery needed for their correction. While previous research has considered acute burn care and the effectiveness of contracture release on functional outcomes, there has been limited work on primary evaluation of burn scars [33–35]. The significant agreement between attending physicians and residents shows the scales effectiveness across levels of surgical training. Nearly three quarters of the burns needing surgical correction would require an advanced reconstructive procedure like a perforator or cross flap. Most patients whose burn scars could benefit from surgical care had just received dressings for their injuries, and less than 15% reported a previous surgery. This outlines the unmet need for surgical burn care in rural communities in Mozambique. In the hands of a trained community health worker, this scale could be implemented to rate burn scars and qualify the degree of surgical complexity necessary for their correction. This could be used to more effectively allocate limited surgical resources in a country that in 2015 had an estimated 25 general surgeons serving a population of more than 29.5 million individuals [16].

This study has several limitations. First, it does not measure the total burden of burns in Mozambique because of its focus on burn scars. As an example, deaths from more severe burns are not captured in our study. Second, because our survey was done almost 10 years ago (2012–2013), we cannot be sure that our results represent the current state of burn care in rural areas of Mozambique. Nevertheless, because few investments have been made in improving surgical care in the more remote areas of Mozambique during the past 10 years, we are confident that our burn data still reflects the current situation in rural areas. Finally, our results are not generalizable to the entire population of Mozambique because rural and urban areas likely differ in burn epidemiology.

In conclusion, this community-based survey provides insight into the prevalence and severity of burn scars in rural Mozambique. This work can inform prevention and treatment strategies in regions where access to medical and surgical care is limited. Future research should work to characterize trends in burn scar incidence and prevention in rural settings and put this novel scale into practice to assess surgical need in a prospective study.

## Supplementary Information

Below is the link to the electronic supplementary material.

Supplementary file1 (DOCX 13 kb)

## Data Availability

The dataset analyzed and the excel spread sheets for the analysis are available upon reasonable request from the corresponding author: pbarba@health.ucsd.edu.
