# Supplementary material for: Prevalence and Severity of Burn Scars in Rural Mozambique
Source: World J Surg. 2022 Aug 10;46(11):2561–9. doi: 10.1007/s00268-022-06682-y (PMC9529692; doi:10.1007/s00268-022-06682-y)
Supplement: Supplementary file 1 — Supplementary file1 (DOCX 13 kb) [file 268_2022_6682_MOESM1_ESM.docx]

| MASCC | |
| --- | --- |
| Definition | Parameter |
| Scar band is thinner than 50% of the joint width | **Narrow** |
| Scar band is equal to or wider than 50% of the joint width | **Wide** |
| Length of the scar band is shorter than the joint’s width | **Short** |
| Length of the scar band is equal to or longer than the joint’s width | **Long** |
| Distance from the original flexion crease to the midpoint of the scar’s free border is less than 50% of the joint's width | **Low** |
| Distance from the original flexion crease to the midpoint of the scar’s free border is equal to or greater than 50% of the joint’s width | **High** |

Burn Groups

Type A - Narrow and Long or Short and Low

Type B - Narrow and Long or Short and High

Type C - Wide and Long or Short and Low

Type D - Wide and Long or Short and Low
